# Dr. Henry J. McKellops

**Published:** 1901-05

**Authors:** 


### DR. HENRY J. McKELLOPS.

A MESSAGE from St. Louis, as we go to press, announces the death of Dr. H. J. McKellops. This will be sad information to Dr. McKellops's many friends throughout the country, for it has been given to but few to be better known or more generously appreciated. Full notice of his life work is deferred until the June number.

